# Suspected drug-induced liver injury associated with iguratimod: a case report and review of the literature

**DOI:** 10.1186/s12876-018-0858-z

**Published:** 2018-08-24

**Authors:** Xiao-li Li, Xiao-Chang Liu, Yu-Lin Song, Ru-Tao Hong, Hai Shi

**Affiliations:** Department of Gastroenterology, First Affiliated Hospital of Anhui Medical University, NO.218, Jixi Road, Hefei, 230022 Anhui Province China

**Keywords:** Drug-induced liver injury, Iguratimod, Clinical features, Pathogenesis, Treatment

## Abstract

**Background:**

Iguratimod is a novel anti-rheumatic drug with the capability of anti-cytokines as report goes. It has been reported that iguratimod is effective and safe for rheumatoid arthritis and other rheumatisms. As side effects, iguratimod can cause gastrointestinal reactions, dizziness, headache and itchy.

**Case presentation:**

In this case report, a 60-year-old female patient was admitted with suspected drug-induced liver injury (DILI) caused by iguratimod. The causality assessment was done by the updated RUCAM, and the possibility of the case in our paper diagnosed as highly probable for the score was 9 points. Iguratimod was discontinued immediately, and methylprednisolone was used for acute liver injury and Sjogren’s syndrome. The data showed the patient has improved gradually, and she was discharged on day 27. The true incidence of iguratimod-related hepatotoxicity and its pathogenic mechanism are largely unknown. It is difficult to recognize and diagnose DILI, and there is no standard for diagnosis of DILI. At the same time, the DILI is still lack of specific treatment.

**Conclusions:**

Based on this rare case of severe liver injury, we recommend careful monitoring of liver function throughout iguratimod treatment for diseases.

## Background

Iguratimod is a member of the family of methanesulfonanilide non-steroidal anti-inflammatory drugs, and it has been reported that iguratimod could cause reversible increase in liver enzymes. DILI is the result of a complex interplay between potentially immunogenic drugs or metabolites and the host’s immune response. DILI is a common liver disease which generally occurs between several days and a few months after drug ingestion. The clinical features of DILI are variable, ranging from transient mild elevation of liver enzymes to fulminant liver failure so as to cause death. With the continuous emergence of new drugs and the insufficient knowledge of their safety in the medical staff and the public, the incidence of DILI has been increasing yearly. However, the diagnosis of DILI is very difficult and is mainly based on circumstantial evidence. Another problem is that DILI is still lack of specific treatment. The treatment of DILI is guided by the degree of hepatic dysfunction and comorbid conditions. And avoiding using the drugs with liver toxicity is the best way to prevent DILI. Post-marketing surveillance study showed that iguratimod could cause increase of aminotransferase. It has been reported one case that iguratimod could cause reversible increase in liver enzymes in China. However, server DILI caused by iguratimod has not been reported.

Herein, we report a case of severe suspected DILI caused by iguratimod in the First Affiliated Hospital of Anhui Medical University and reviewed the papers related to DILI in the literature.

## Case presentation

**Fig. 1:**
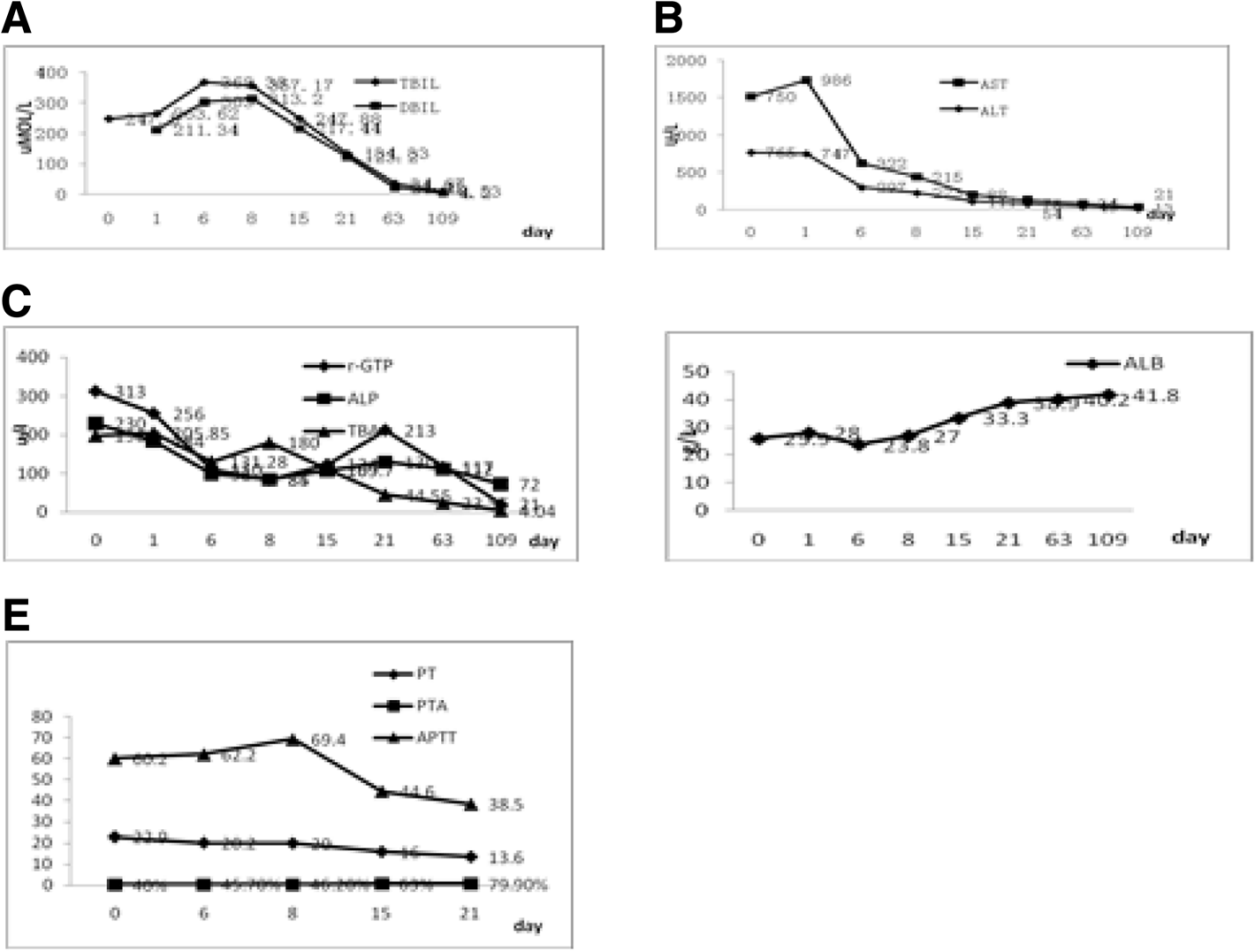
included five pictures, respectively (**a**, **b**, **c**, **d** and **e**), which mainly showed the trends of some laboratory test results of this patient. For example, **a** mainly showed the changes of TIBL and DIBL during the course of the disease; **b** represented the changes of ALT, AST in the course of disease; **c** mainly showed the changes of r-GT, ALP and TBA during the course of the disease; **d** represented the changes of ALB in the course of disease; And E mainly showed the changes of PT, APTT and PTA during the course of the disease

A 60-year-old female patient was admitted to our hospital on October 28th, 2016 with the symptoms of abdominal pain, distension, dark urine, cough, expectoration, chills and fever. The highest temperature was 39 °C before her admission. She had been taking iguratimod (25 mg twice per day) because of Sjoren’s syndrome (SS) for about 15 days prior to her admission. The patient didn’t have hepatobiliary disease and history of excessive alcohol intake, recent travel, blood transfusion. According to the physical examination, her vital signs were normal. Despite sever jaundice, she was conscious. There was no bleeding points or spider angioma or liver palm on her skin. Her abdomen was flat and soft, with no tenderness or rebounding tenderness. Additionally, her liver and spleen were untouched, without shifting dullness. Besides, no edema was seen in her entire body. Her blood test results (Table 1) were as follows: complete blood count: WBC 3.54 × 10^^9^/L, NE 61.00%, Hb 119 g/L, PLT 130 × 10^^9^/L, PT 22.9 s, APTT 60.2 s, PTA 78%. Abnormal liver tests: TBIL 263.62 umol/L, DBIL 211.34 umol/L, IBIL 52.28 umol/L, ALT 747 U/L, AST 986 U/L, gamma-GPT 256 U/L, ALP 184 U/L, TBA 205.85 umol/L, LDH 346 U/L. The serum IgG was 13.68 g/L, and the level of IgG4 was 298 μg/ml. The patient was negative for IgM anti-HA, anti-HCV, anti-HEV, HBsAg, anti-EBV-VCA IgM. The serologic markers of hepatitis B virus HBsAb, HBcAb, HBeAb were positive, but the quantification of hepatitis B virus was normal. The results of ANAs were as follows: ANA (positive, nuclear particle type 1:3200), anti-SS-A (60) antibody (positive), anti-SS-A (52) antibody (positive), anti- La/SS-B antibody (positive). The results of autoimmune hepatitis markers (AMA), ANCA, ACL, anti-O antibody, rheumatoid factor, thyroid function and T-SPOT test were also normal. And phlegm etiology detection, blood culture, G and GM tests were normal. The serum C3 and C4 were normal. The CRP and PCT were moderately elevated which the highest level of CRP and PCT was 10.87 mg/L and 1.780 ng/ml, respectively. The result of ECG was normal. Abdominal ultrasound scan showed cholecystitis and ascites. The chest computed tomography (CT) found that bilateral pleural effusion and pneumonia at the lower right on November 31th, 2016. After 1 week, the review of chest CT revealed that bilateral pleural effusion was absorbed a little compared with the last result, and pneumonia was still existed. Combined with the history of related medication, symptoms, signs and the relative auxiliary examination, the patient was diagnosed as likely to be suffering from DILI, Sjogren’s syndrome, pneumonia, and hypoproteinemia. However, considering the patient with Sjogren’s syndrome and the higher level of ANA, antoimmune liver injury couldn’t be ruled out. According to the International Autoimmune Hepatitis Group’s (IAIHG) AIH diagnostic scoring system, the patient was rated 6 points and the AIH could be ruled out basically. To confirm the diagnosis, liver biopsy was proposed, but the patient refused due to economic reasons.Taking into account of the long-term use of hormones in patient, the routine blood and inflammation index couldn’t reflect the severity of infection, and the patient was given the treatment such as controlling infection, liver-protecting, albumin replenishing and other supporting treatments. However, the symptoms such as jaundice, abdominal distension increased and fever still appeared intermittently, while cough and expectoration have been improved obviously. And review of the relevant indicators (Fig. 1) prompted that serum bilirubin level increased, transaminase and albumin level decreased and blood coagulation declined further. The above indicators suggested that abnormal liver function has reached to a higher level. The patient was given the treatment like infusion plasma to improve the function of blood coagulation. At the same time, the anti-infection therapeutic regimen was strengthened. On the 7th day of admission, we conducted a peritoneal puncture and checked the relevant indicators, and finally, the results suggest that the diagnosis of spontaneous peritonitis was ruled out. In order to clarify the cause and guide the next treatments, a multidisciplinary discussion was organized. Then the treatment was adjusted as follows: methylprednisolone was taking to control Sjogren’s syndrome, and we could use hormone via statics drop when the fever occurred again. About one month after given up taking iguratimod, the patient’s symptoms improved significantly, and the relevant indicators such as abnormal liver tests, blood coagulation function returned to normal basically (Fig. 1). According to the updated RUCAM diagnostic scoring system, the patient was rated 9 points and was a suspected DILI. After discharged, the patient was re-adjudicated a one-month follow-up by the same reviewers and the diagnosis was confirmed.

**Table 1:** Mainly described some of the laboratory tests during the hospitalization of this patient

WBC(*10^^^9/L)	3.54	Albumin(g/L)	28.0	ANCA	negative
Neutrophils(%)	61.00	TBIL(umol/L)	263.62	ACL	negative
Lymphocytes(%)	29.70	DBIL(umol/L)	211.34	AMA-M	negative
Eosinophils(%)	0.60	IBIL(umol/L)	52.28	HBsAg	negative
Basophils(%)	0.80	AST(U/L)	747	HBsAb	positive
RBC(*10^^^12/L)	3.93	ALT(U/L)	986	HBeAb	positive
Hemoglobin(g/L)	119	ALP(U/L)	184	HbcAb	positive
Platelets(*10^^^9/L)	130	r-GTP(U/L)	256	HBV-DNA(IU/ml)	< 20
PT(S)	22.9	LDH(U/L)	346	anti-HA	negative
APTT(S)	60.2	TBA(umol/L)	205.85	anti-HCV	negative
PTA(%)	40.0	IgG4(ug/ml)	265	anti-HEV	negative
CRP(mg/L)	6.23	IgG(g/L)	13.68	anti-HSV	negative
PCT(ng/ml)	0.56	IgA(g/L)	2.41	anti-CMV IgM	negative
G test	negative	IgM(g/L)	2.62	anti-CMV IgG	positive
GM test	negative	complementC3(g/L)	0.26	anti-EBV VCA IgM	negative
Blood culture	negative	complement C4(g/L)	0.07		
T-SPOT test	negative	ANA	positive,1:3200		
RF	normal	anti-SS-A(60)	positive		
Anti-O	negtive	anti-SS-A(52)	positive		
		anti-La/SS-B	positive

## Discussion and conclusion

DILI is caused by various prescription or non-prescription chemical drugs, biological agents, traditional Chinese medicine (TCM), natural medicine, dietary supplements, their metabolites and even excrement [1, 2]. DILI is very common, and it is one of the most serious adverse drug reactions (ADRs). And in some critical patients, it can lead to acute liver failure (ALF) or even death [3]. According to the statistical results, there are more than 1100 kinds of drugs that can induce to DILI at present, among which the highest incidence of DILI is caused by antibacterial, anti-tuberculosis, chemotherapy, and Chinese herbal medicine [2, 4].

Iguratimod (T614,3-formylamino-7-methylsulfonylamino-6-phenoxy-4H-1-benzopyran-4-one), a small-molecule compound, was developed as a disease-modifying antirheumatic drug in Japan. The pharmacological studies showed that inhibition of the production of cytokines and immunoglobulins mainly contributes to its improvement effect on animal arthritis models. A large number of clinical studies have confirmed the real-world safety and effectiveness of iguratimod. In Japan, it became available in 2012 [5]. Later, iguratimod was shown to be effective in patients with Sjogren’s syndrome. However, it also can cause some side effects such as gastrointestinal reactions, dizziness, headache, itghy, drowsiness and aminotransferase increase [6, 7]. In order to determine the safety and effectiveness of iguratimod for RA, a 52-week post-marketing surveillance study was conducted in 2769 patients in Japan. The results showed that the overall incidences of adverse events (AEs), adverse drug reactions (ADRs) and serious ADRs were 38.41, 31.65 and 3.21%, respectively. The most commonly reported serious ADRs were pneumonia / bacterial pneumonia, interstitial lung disease and Pneumocysis jiroveci pneumonia [8]. But, it has not been reported that severe DILI caused by iguratimod.

With the continuous emergence of new drugs and the insufficient knowledge of their safety in the medical staff and the public, the incidence of DILI has been increasing yearly. And the morbidity of DILI is estimated at between 1/100000 and 20/100000 or less in developed countries [9]. The acute DILI accounts for about 20% of hospitalized patients with acute liver injury in China. The incubation period is very different, ranging from a few days to several months. The clinical features of DILI are also usually non-specific. In most patients, they only manifest the liver biochemical indexes increased in varying degrees such as serum ALT, AST, TBIL, DBIL and ALP. Many patients might manifest some unspecific features such as fatigue, disgusted with oil, liver pain and epigastric discomfort. Besides, the patient with obvious cholestasis might manifest jaundice, itchy and dark urine.

DILI can be classified into 2 types according to the pathogenesis: intrinsic DILI and idosyncratic DILI (IDILI). One of the characteristics of IDILI is that it occurs rarely and only in a subset of individuals with a presumed susceptibility to the drug. According to the pattern of liver tests observed, there are 3 types of IDILI: hepatocellular, cholestatic and mixed, which was proposed by the Roussel Uclaf Causality Assessment Method (RUCAM) [10, 11]. There are few clinical features specifically associated with DILI. As just mentioned, DILI can vary from a mild transient elevation of ALT and AST, usually asymptomatic, to acute hepatitis or even liver failure. Although fever, rash and arthralgia are symptoms and signs of an immunoallergic reaction to a drug, they also be seen without taking any drugs and the frequencies in patients with DILI are not high. The risk of factors for DILI is influenced by multiple, such as drug regimen, gender, age, malnutrition, diabetes, alcohol drinking and hepatitis B or C chronic infection. Moreover, genetic factors for drug metabolism, such as polymorphisms of cytochrome P (CYP) 450 or deficiency of N-acetytransferse, have also been reported to contribute to DILI.

Since there are no diagnostic tests or specific biomarkers for DILI, its diagnosis is made after stringently excluding other causes of liver disease, including viral and autoimmune hepatitis, bile duct obstruction, hepatic ischemia, sepsis and metabolic disorders. As there are no criteria of diagnosis for DILI, many clinical scales have been developed. In the early 1990s, the diagnosis scale called Roussel Uclaf Causality Assessment Method (RUCAM) was proposed [12]. The diagnosis of DILI by the first edition of RUCAM was mainly based on classification of clinical, biochemical and serological features and non-drug factors. It had been effectively validated in positive reexploding experiments and had become a global standard. At the same time, the first edition of RUCAM also has many shortcomings, such as: the unclear and incomplete definition of certain core elements and structural words, complicated evaluation interface, and so on. Considering the reasons above, the main makers of the first edition of RUCAM proposed an updated plan for the diagnostic criteria of RUCAM [11]. The causal correlation of drugs and liver injury is divided into 5 levels according to the updated RUCAM: highly probable: score > 8; probable: 6 ≤ score ≤ 8; possible: 3 ≤ score ≤ 5; unlikely: 1 ≤ score ≤ 2; excluded: score ≤ 0. Although various causality assessment tools exist for DILI, the updated RUCAM is the most commonly used [11]. Combined with the above, the diagnosis of DILI must rely on detailed medical history and all clinical data, including symptoms, laboratory and imaging examination results. When necessary, we can conduct a liver biopsy.

In our case, the patient was a 60 years old woman. She had been taking iguratimod (25 mg twice per day) because of SS for about 15 days prior to her admission. Later, the symptoms such as abdominal distention, jaundice, dark urine and disgusted with oil appeared gradually. And the viral and autoimmune hepatitis, bile duct obstruction, hepatic ischemia and metabolic disorders were excluded. According to the IAIHG AIH diagnostic scoring system [13], the patient was rated 6 points and the AIH could be ruled out basically. According to the standards proposed by the updated RUCAM [11], the type of DILI was hepatocellular because the alanine aminotransferase (ALT) > 3 times upper limits of normal (ULN) and *R* ≥ 5, where R is the ratio of serum activity of ALT / serum activity of alkaline phosphatase (ALP), both of which are expressed as multiples of the ULN. The possibility of the case in our paper diagnosed as DILI were highly probable for the score was 9 according to the updated RUCAM [11]. In addition, the patient was re-adjudicated a one-month follow-up by the same reviewers and the diagnosis was confirmed.

Understanding the pathogenesis of DILI has greatly advanced in recent years largely benefit from genetic studies and improved animal models. DILI can occur as a result of dose related direct drug toxicity, as seen with acetaminophen, mitochondrial poisons, or certain chemotherapy drugs. On the other hand, the majority of DILI due to many drugs occurs in only a small proportion of patients and is therefore considered idiosyncratic due to individual susceptibility. The pathogenesis of DILI is complex, involved in drug metabolism, mitochondrial function damaged, immune response, genetic and environmental. Among the various drug metabolizing enzymes, cytochrome P450s (CYP450) constitutes an important protein family that aside from functioning in xenobiotic metabolism is also responsible for a diverse array of other roles encompassing steroid and cholesterol biosynthesis, calcium homeostasis and growth regulation. About 90% of drugs is metabolized by CYP450, some of them can induce or inhibit the activity of CYP450 to strengthen or weaken its efficacy. For example, Rifampicin is an inducer of CYP450, which can accelerate the CYP enzymes and enhance its activity, and then speed up their own and other drugs metabolismed. Immune response is also an important pathogenesis of DILI. High mobility group box-1 (HMGB1) is a later inflammatory mediator, which is an important marker of immunological damage to DILI [14]. HMGB1 can be served as an important, simple and predictable marker of the DILI. The pathogenesis of DILI is not very clear. In order to prevent the emergence of DILI, the pathogenesis of DILI needs further research.

Iguratimod is a member of the family of methanesulfonanilide non-steroidal anti-inflammatory drugs (mNSAIDs), most of which act as cyclooxygenase (COX)-2 inhibitors. It can play a role in rheumatisms mainly by inhibiting the production of inflammatory factors such as IL-1, IL-6, IL-8 and TNF-a, improving bone metabolism [15, 16]. It yields a strong improvement in arthritis via exact suppression of receptor activator of nuclear factor kB ligand (RANKL) / osteoprotegerin, IL-17 and MMP-3 expression in synovial fibroblasts from rheumatoid arthritis patients [17]. In vivo, iguratimod can be divided into two major forms of M1 and M2. M1 and M2 have the ability to reduce the cytotoxicity of leucine methyl ester [18]. Preclinical plarmacokinetic studies have shown that iguratimod raw materials are in line with the first absorption in animals. The total excretion of the protoplast in the feces, urine and bile acid is less than 20%. At present, iguratimod has been widely used in patients because of its effectiveness and safety. However, although not common, iguratimod also can induce some ADRs such as gastrointestinal reactions, dizziness and aminotransferase increase. Unfortunately, the mechanism of action and leading to ADRs have not yet been clarified. Therefore, it is very important to further study iguratimod-related hepatotoxicity and its pathogenic mechanism.

Up to now, DILI is still lack of specific treatment. The treatment of DILI is guided by the degree of hepatic dysfunction and comorbid conditions. Patients should be especially cautious about using drugs, and inform their doctor about any drugs or other substances they are taking, including prescription and over-the-counter medications, recreational drugs. The patient with liver dysfunction, the elderly and children reduce the dose of drugs with liver toxicity. In the event of DILI, especially towards 3 to 5 times’ increase in serum transaminases, hepatotoxic drugs should be stopped immediately. N-acetylcysteine (NAC) is the only effective detoxification drug approved by U.S. FDA in 2004 for the treatment of acetaminophen (APAP) – induced intrinsic DILI [19]. Lots of clinical experiments conformed that NAC can remove a variety of free radicals, the sooner, the better. It has been proposed that the magnesium isoglycyrrhizinate can be used to treat acute hepatocellular or mixed DILI with significantly elevated ALT in September 2014 [20]. Ursodeoxycholic acid can be used to treat cholestatic DILI, and adenosylmethionine is also benefit to cholestatic DILI [21]. And glucocorticoids are recommended for the treatment of DILI with acute liver failure (ALF), but lack of evidence support. For the patient with hepatic encephalopathy, severe coagulation dysfunction in ALF, and decompensated cirrhosis, liver transplantation can be taken.

DILI is caused by various prescription or non-prescription chemical drugs, biological agents, and its metabolites. The incidence of DILI has been increasing yearly. And the clinical manifestations and incubation period of DILI are usually non-specific. A large number of studies have found that DILI is the result of a variety of pathogenic factors, such as age, gender, gene and environment [22]. The diagnosis of DILI is often challenging, and there is no clear standard for diagnosis of it at present. The diagnosis of DILI should rule out other causes such as viral and autoimmune hepatitis, bile duct obstruction, metabolic disorders. DILI is still lack of specific treatment, and the best way to prevent DILI is to avoid using the drugs with liver toxicity. Therefore, as a doctor, we should guard against the emergence of DILI when the patient is taking some suspicious drugs. The patient should detect liver function, routine blood and other indicators on a regular basis to prevent the occurrence of DILI.

## Abbreviations

ADRs: Adverse drug reactions
AEs: Adverse events
ALF: Acute liver failure
ALP: Alkaline phosphatase
ALT: Alanine aminotransferase
AMA: Antimitochondrial
ANA: Antinuclear antibodies
anti-CMV: Cytomegalovirus antibody
anti-EBV VCA: Anti-Epstein-Barr virus viral capsid antigen antibody
anti-HA: Hepatitis A antibody
anti-HCV: Hepatitis C virus antibody
anti-HEV: Hepatitis E virus antibody
APTT: Activated partial thromboplastin time
AST: Aspartate aminotransferase
CRP: C-reactive protein
DBIL: Direct bilirubin
DILI: Drug-induced liver injury
HMGB1: High mobility group box-1
IDILI: Idiosyncratic drug-induced liver injury
LDH: Lactate dehydrogenase
NADRPS: The Naranjo Adverse Drug Reactions Probability Scale
PCT: Procalcitonin
PT: Prothrombin time
PTA: Prothrombin activity
RBC: Red blood cell
RF: Rheumatoid factor
r-GTP: Gamma-glutamyltranspeptidase
RUCAM: Roussel Uclaf Causality Assessment Method
SS: Sjoren’s syndrome
TBA: Total bile acid
TBIL: Total bilirubin
TCM: Traditional Chinese medicine
ULN: Upper limits of normal
WBC: White blood cell
